# Systematic analysis of prophage elements in actinobacterial genomes reveals a remarkable phylogenetic diversity

**DOI:** 10.1038/s41598-023-30829-z

**Published:** 2023-03-17

**Authors:** Vikas Sharma, Max Hünnefeld, Tom Luthe, Julia Frunzke

**Affiliations:** Institute of Bio- and Geosciences (IBG-1) Biotechnology, Forschungszentrum Jülich, 52425 Jülich, Germany

**Keywords:** Phage biology, Bacterial genomics

## Abstract

Actinobacteria represent one of the largest bacterial phyla harboring many species of high medical, biotechnological and ecological relevance. Prophage elements are major contributors to bacterial genome diversity and were shown to significantly shape bacterial fitness and host-microbe interactions. In this study, we performed a systematic analysis of prophage elements in 2406 complete actinobacterial genomes. Overall, 2106 prophage elements were predicted to be present in about 50% (1172/2406) of the analyzed datasets. Interestingly, these identified sequences compose a high prevalence of cryptic prophage elements, indicating genetic decay and domestication. Analysis of the sequence relationship of predicted prophages with known actinobacteriophage genomes revealed an exceptional high phylogenetic diversity of prophage elements. As a trend, we observed a higher prevalence of prophage elements in vicinity to the terminus. Analysis of the prophage-encoded gene functions revealed that prophage sequences significantly contribute to the bacterial antiviral immune system, but no biosynthetic gene clusters involved in the synthesis of known antiphage molecules were identified in prophage genomes. Overall, the current study highlights the remarkable diversity of prophages in actinobacterial genomes, with highly divergent prophages in actinobacterial genomes and thus provides an important basis for further investigation of phage-host interactions in this important bacterial phylum.

## Introduction

The phylum of Actinobacteria belongs to one of the largest taxonomic units of Bacteria comprising species of high medical, ecological and biotechnological relevance^1^. Most members are free-living species thriving in soil (*Streptomyces, Mycobacteria,* and *Corynebacteria*) or marine (e.g., *Streptomyces, Micromonospora, Rhodococcus,* and *Salinispora* species) environments, but the phylum is also renowned for several prominent human or animal pathogens, like *Mycobacterium tuberculosis* or *Corynebacterium diphtheriae*. Currently, about two-thirds of the antibiotics used in the clinics are derived from soil-dwelling *Streptomyces* species^1^. Furthermore, pathogenic bacterial species such as *M. tuberculosis* cause high morbidity and mortality to humans. The evolution of multi-drug-resistant mycobacterial strains is a major public health concern and economic threat worldwide^2^. Gain of such resistance traits has frequently been reported from horizontal gene transfer events from other pathogenic bacteria via mobile genetic elements such as plasmids, transposons, and phages^3^.

Bacteriophages (short phages) are viruses infecting bacteria. They typically follow one of two different phage lifestyles: the lytic or the lysogenic cycle. Upon infection, virulent phages reprogram the host to produce new virions leading to cell death through cell lysis. In contrast, temperate phages may opt for the integration into the chromosomal DNA of the host and stay in a dormant state replicating as a so-called prophage in concert with the host genome. In this state, the evolutionary fates of phage and host are aligned. It is therefore not surprising that prophages can equip their hosts with traits involved in phage defense, stress tolerance or virulence factors^4–7^.

Prophage elements are known to account for a significant fraction of strain-specific differences within species and may comprise up to 20% of the total bacterial genomic DNA^8,9^. After integration, prophage DNA can undergo rapid genetic decay such as genomic rearrangements, mutation, insertion, duplication, or deletions^10,11^. As a result of this prophage ‘domestication’ a large fraction of prophages in bacterial genomes are defective (remnant) at several levels, including cell lysis, infectivity or virion assembly^12,13^.

Upon stress conditions, prophages can revert to the lytic cycle, thereby posing a threat to their host^14^. However, spontaneous prophage induction can also lead to a competitive advantage for the host, for example by promoting biofilm formation, the release of bacterial toxins or facilitating horizontal gene transfer^15,16^. While several studies focused on the impact and interaction of prophages in proteobacterial strains, no systematic study on the distribution of prophages in actinobacteria has been performed up to now.

Insights into the genomic diversity of phages infecting actinobacteria (actinobacteriophages) has increased dramatically over the past few years. The impressive effort of research and education-oriented programs such as the ‘Phage Hunters Integrating Research and Education’ (PHIRE) or ‘Science Education Alliance Phage Hunters Advancing Genomics and Evolutionary Sciences’ (SEA-PHAGES) has yielded in the discovery and sequencing of diverse actinobacteriophages (> 20,000 isolates as of spring 2022)^17,18^. Out of these 20,000, more than 4000 temperate and virulent phages are completely sequenced. However, this dataset includes more than 50% of sequences from a single host genus, *Mycobacterium*^17–19^*.* Actinobacteriophages (phages infecting actinobacteria) are typically linear dsDNA (double-stranded DNA) viruses with a genomic size ranging from 14 to 194 kbp^20,21^. Most of these isolated viruses are described as tailed-phages belonging to three viral families (*Myoviridae, Podoviridae*, and *Siphoviridae*)^18,21,22^. However, also non-tail *Tectiviridae* phages are reported in the literature^23^.

Comparative analysis of actinobacteriophages displays a high genomic diversity due to recombination and horizontal gene transfers between the hosts and related viruses and revealed a high gene content flux within temperate phages compared to virulent phages^22^. Typical for phage genomes, actinobacteriophages encode a significant amount of “dark matter” or hypothetical proteins which do not share similarities with genes of known function available in public databases^20,21,24^. So far, based on shared genomic features and complete nucleotide sequence similarity, actinobacteriophages are divided into more than 27 clusters and more than 100 sub-clusters. However, several sequences remain ungrouped due to a lack of similar genomes^18,21,22^.

In this study, we conducted a large-scale analysis of putative prophage elements in actinobacterial genomes. Insights gained on the prevalence of prophages in actinobacteria provide a basis to understand their contribution to host evolution and physiology.

## Results and discussion

### Prevalence of prophage-like sequences across actinobacterial genomes

In order to determine the prevalence of prophage sequences within actinobacterial genomes, we screened 2406 complete genomic sequences from actinobacterial strains using two different programs (Virsorter2^25^ and VIBRANT^26^) with default parameters. While the resulting analysis with Virsorter2^25^ led to the discovery of 1806 putative prophage-like sequences from 45% (1100/2406) of total analyzed genomes, VIBRANT^26^ showed a slightly higher prevalence and predicted 2112 putative prophages present in 48% (1172/2406) of the analyzed bacterial genomes (Supplementary Table S1). Comparing the results of the two prediction tools showed that more than 75% (Virsorter2: 902/1100, Vibrant: 902/1172) of bacterial genomes encoding prophages were commonly predicted by both tools. Each tool also discovered less than 24% (Virsorter2: 198/1100, Vibrant: 270/1172) of unique prophage encoding genomes. The identified prophage prevalence comparison revealed similar or slight differences in frequency distribution according to host genus (Supplementary Fig. S1). Using VIBRANT, we were able to identify a large cryptic prophage element in the *Corynebacterium glutamicum* strain ATCC 13,032, which is in line with previous experimental results^27,28^. In contrast, Virsorter2^25^ completely missed this sequence and has recently been found to be less effective^29^ than other prophage prediction tools, including VIBRANT^26^, PhiSpy^30^, and Phigaro^31^. Moreover, we also validated if our findings are comparable with the previously published studies on the discovered prophages present in *M. abscessus genomes*^16,32,33^*.* The resulting analysis showed high accordance in terms of the number of identified prophage sequences by our analysis using VIBRANT as compared to the DEPhT tool recently described by Gauthier et al.^33^ (Supplementary Table S2). As previously reported^33^, DEPhT discovered some non-reference extra prophages in two *M. abscessus* genomes (CP065287, CP065265). Similarly, we could also find additional sequences in the three bacterial genomes (CP065287, CP063320, CP065273). However, we found differences in the prophage coordinates compared to the reference genomes. The observed positions mainly show coordinate overlapping, comparable to previous findings^33^ (Supplementary Table S2), suggesting the differences occurred due to different prediction programs and associated background tools and databases. Nevertheless, it is important to note that we did not miss any prophage sequence in our analysis compared to the reference dataset used in Gauthier et al. 2022 publication^33^. These results show that the results of different tools naturally differ from each other and can also contain false predictions. A problem which can only be resolved by experimental analyses.

**Figure 1:**
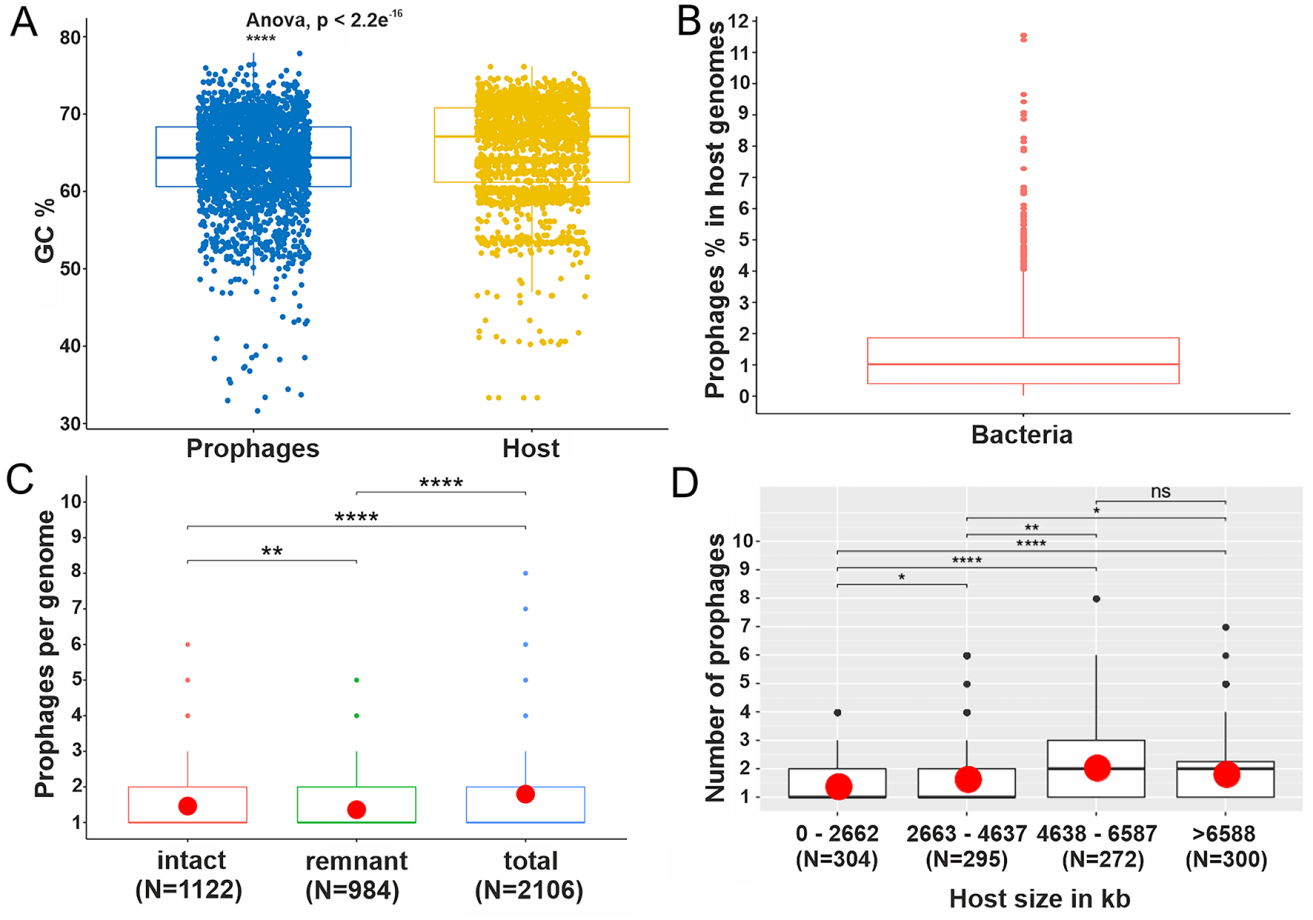
Distribution of prophages in actinobacterial strains. (**A**) GC content comparison between identified prophage elements and their host genomes. Significance levels are shown based on *p* values (****, *p* < 0.0001). (**B**) Percentage of prophage elements in actinobacterial chromosomes. (**C**) The box plot represents the average number of prophages per actinobacterial genome categorized as "intact" and "remnant" elements. The red circle highlights the mean value. Significance levels based on wilcoxon test are shown on the basis of *p *values (ns, *p* > 0.05; *, *p* < 0.05; **, *p* < 0.01; ****, *p* < 0.0001). (**D**) Distribution of prophages according to the size of the bacterial genome. The boxplot represents the average number of prophages within different bins containing different host genome sizes. The red circle highlights the mean value across the assigned groups.

Overall, these validations showed that the VIBRANT program is a reliable tool for prophage prediction. Therefore, the results obtained using Virsorter2 were disregarded for further analysis and we focused entirely on the VIBRANT estimations. Following these results, we detected 2112 putative prophages-like sequences across 75% (152/203) of the analyzed host genera within the phylum of Actinobacteria. Considering the sequencing bias, it is not surprising that most prophage encoding strains belong to the genera of *Mycobacterium* (26%; 138/520), *Corynebacterium* (42%; 138/325), *Streptomyces* (62%; 196/314) and *Bifidobacterium* (80%; 149/183). In contrast, several bacterial strains with few representatives (1–4 genomes) belonging to 51 genera do not encode any prophage (Supplementary Table S1, Sheet3). The identified prophage sequence size ranges from 694 to 254,681 base pairs and up to 8 prophage elements were detected in actinobacterial genomes in our analysis (Supplementary Table S1, Sheet1; Supplementary Table S3, Sheet1). Members of the actinobacteria cover a large range with respect to their genomic GC content, which is also reflected by their prophage sequences ranging between 31 to 78%, which is on average lower than that of their host genomes (Fig. 1A).

Next, we calculated the overall prophage fraction according to the length of total host genome. Except for two *Propionibacterium freudenreichii* chromosomes (LT618778.1, LT618794.1), the prophage fraction lies between 0.01 to 11% of the total bacterial host genome, which is in line with previous findings (Fig. 1B)^9^. These two exceptional *Propionibacterium* sequences represent a 100% prophage fraction in host genomes. Each of these sequences shares a significant similarity (> 90%) with temperate *Propionibacterium freudenreichii* phages (NC_031108.1, KX620754.1) belonging to different clusters (BX, BW) (Supplementary Tables S1, S3, S4, Sheet1) indicating the discovery of mis-annotated viral sequences from the NCBI database.

### Distribution of intact and remnant prophages in actinobacterial genomes

In order to gain insights into the nature and origin of the identified prophages, all 2112 sequences (Supplementary Table S3) were annotated based on conserved protein domains using RPS-TBLASTN against the CDD database^34^. Based on this analysis, we discarded six short sequences that showed no conserved domains at all. Of the remaining 2106 annotated prophages, 53% (1122) were classified as intact based on the presence of an integrase domain and at least one structural domain assigned to capsid, tail or portal proteins (see “Methods” and Supplementary Table S11, Fig. 1C). The remaining 47% (984/2106) of prophage sequences were classified as remnant either, because initially no integrase domain was found or there was no additional structural domain detected. These results suggest a high prevalence of remnant prophages in actinobacterial genomes, which is in line with previous studies focusing on enterobacterial genomes^10^ or prophage elements in the prominent pathogen *Acinetobacter baumanii*^35^. While we used similar criteria for the definition of “remnant” prophages as previous studies^10^, thorough experimental investigation of their inducibility and the formation of infectious phage particles will be required to deduce whether elements are indeed cryptic and to elucidate remaining functions and a potential physiological relevance.

It can be assumed that a significant proportion of these elements—especially the smaller elements (< 15 kbp)—could belong to the group of so-called phage satellites or PICIs (phage-inducible chromosomal elements). Current studies show that these elements are widespread in bacterial genomes and that they significantly contribute to antiviral defense^36,37^. A large body of research on these elements exists, particularly for *Staphylococcus aureus*, but experimental validation for actinobacteria is lagging behind^38^.

Next, we investigated if the actinobacterial strains with bigger genomic sizes allow more prophage integration events. Therefore, we equally divided overall bacterial genomic size into four bins and calculated the average number of prophages in the corresponding genomes. Interestingly, up to 4638 − 6587 kb, the host bacteria showed an increasing trend of harboring more prophages, whereas, after this, we observed no further significant increase in the larger genomes (Fig. 1D).

### Plasmid prophages

Phage-plasmids have recently been identified as a considerable fraction of sequenced plasmids^39^. In this study, we detected 151 prophages within 15% (130/844) of analyzed actinobacterial associated plasmid genomes (Supplementary Table S1, Sheet2). The predicted prophages range from one to three different “prophage elements” per plasmid genome, and their genomic size ranges between 4444 and 425,417 base pairs (Supplementary Tables S1, S3, Sheet2). Compared to bacterial chromosomes, the prophage percentage across the plasmid genomes showed high variability between 0.5 and 100% (Supplementary Table S1, Sheet1–2). Interestingly, more than fifty percent of the plasmids harbouring prophage sequences (78/130) showed more than 50% of prophage fraction in analyzed host genomes, suggesting the potential discovery of phage-plasmids (PP)^39,40^. Comparing our results to previous studies, we confirmed the presence of at least 12 PP, including the misannotated *Propionibacterium freudenreichii* sequence mentioned above (LT618794) (Supplementary Table S5). However, we missed two sequences (CP015530, CP023977) because the analyzed genomic sequences do not encode a sufficient number of hallmark phage-like genes (integrase and genome replication proteins)^39^.

Analysis of the 20 most abundant Pfam domains revealed the presence of genes typically found in phages (Integrase: pfam00589, pfam00665; Cro/C1-type: Pfam01381) and plasmids (ParA/CbiA: pfam01656; ParB: pfam02195) (Supplementary Fig. S2). Overall, these findings confirm the key genomic features of PP like elements and their hidden diversity in actinobacterial plasmid genomes.

### Genomic comparison reveals a remarkable phylogenetic diversity of prophages

Next, we analyzed the relationship of the identified prophages by comparing their sequences with the known actinobacteriophage genomes based on average nucleotide identity using a clustering approach. The resulting comparative analysis with 5377 sequences (2263 prophages obtained from actinobacteria and their plasmids and 3433 phage genomes) allowed us to divide the sequences into 1961 clusters (Supplementary Table S4). Only 1.3% (29/2263) of total identified prophage sequences fall within 17/1961 clusters showing significant sequence relationship with already known actinobacteriophages. However, more than 50% (1243/2263) of the prophages remain ungrouped as a single sequence; 43% (991/2263) of the remaining prophage sequences form clusters (two or more sequences) with themselves but without known actinobacteriophages. These results underline the incredible diversity of prophages within actinobacterial genomes.

**Figure 2:**
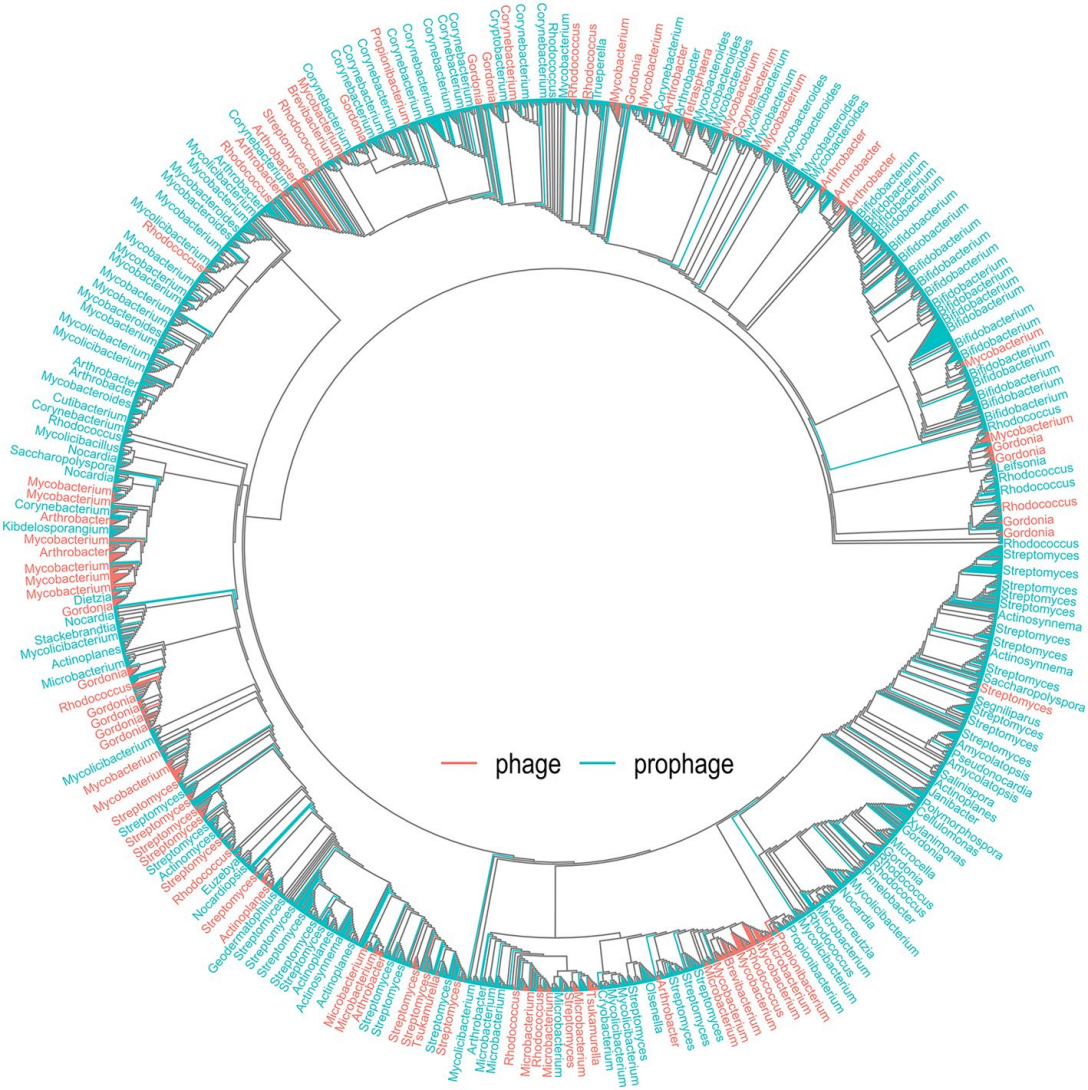
Sequence relationship between prophages and known actinobacteriophages. The k-mer clustering-based phylogeny was constructed using 2704 genomic sequences from prophages (blue) and actinobacteriophages (phages).

In addition, we used an alternative approach to establish the phylogenetic relationship between prophage and actinobacteriophage sequences based on genome-wide distribution of k-mer clustering. Instead of using all 3433 actinobacteriophages genomes in the analysis, the sequence dataset was initially clustered at phage species-level diversity using the clustering approach as mentioned above. Subsequently, a single representative sequence from the resulting 441 clusters, including 2263 prophages was used for the phylogenetic analysis. The resulting unrooted k-mer clustering tree showed the major distribution of prophages specific clades apart from the known phages, indicating the high diversity of viral sequences in actinobacterial genomes (Fig. 2). Again, few prophage sequences form clades together with actinobacteriophage sequences, which is congruent with the clustering analysis (Supplementary Table S4). Altogether, both approaches showed the great diversity of prophages in actinobacterial genomes. Consequently, the genome analysis of intact and cryptic prophage elements provides important complementary data to understand phage diversity and biology in this large phylum.

**Figure 3:**
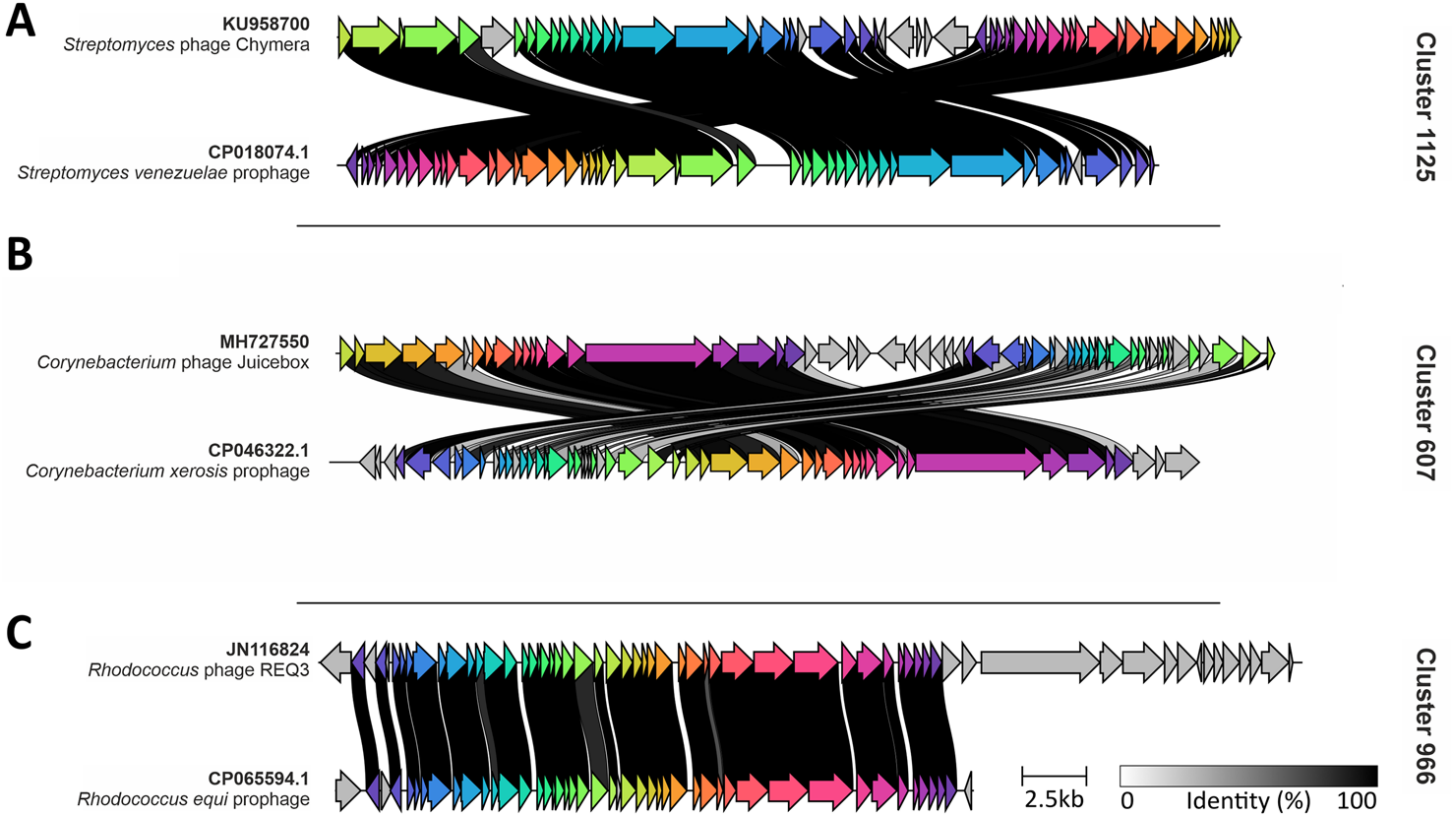
Syntenic analysis between prophages and actinobacteriophage genomes. (**A**–**C**) Shared genes between the prophages and known actinobacteriophage genomes. Arrows of same color indicate genes that are shared in the genomes. The grey horizontal bar represents the level of similarity between the genes across the compared genomes.

After revealing the exceptional prophage diversity, we further looked into selected prophage elements, which show a significant similarity to known actinobacteriophages (Supplementary Table S4, Fig. 3). In most cases, we observed a highly conserved, syntenic organization between the phage and prophage genomes with a certain rearrangement of gene blocks. For example, Cluster_1125 associated with *Streptomyces venezuelae* strain *ATCC 10,712* showed high similarities between the phage Chymera and a remnant prophage genome. Similarly, a prophage of *Corynebacterium xerosis* (CP046322.1_fragment_7) belongs to Cluster_883 and shares a high similarity with a phage infecting *Corynebacterium xerosis* (MH727550). Besides that, a genomic rearrangement was observed in *M. tuberculosis* prophages (CP072761.1_fragment_46_1, CP072762.1_fragment_46_1) (Supplementary Fig. S3). However, these non-bonafide phage elements were integrated into the host bacterial genomes to develop phage-resistant mutants^41^.

In contrast, *Rhodococcus* intact prophage belonging to Cluster_966 shows a high collinearity compared to the known *Rhodococcus* phage REQ3.

### Prophage integration sites in actinobacteria

**Figure 4:**
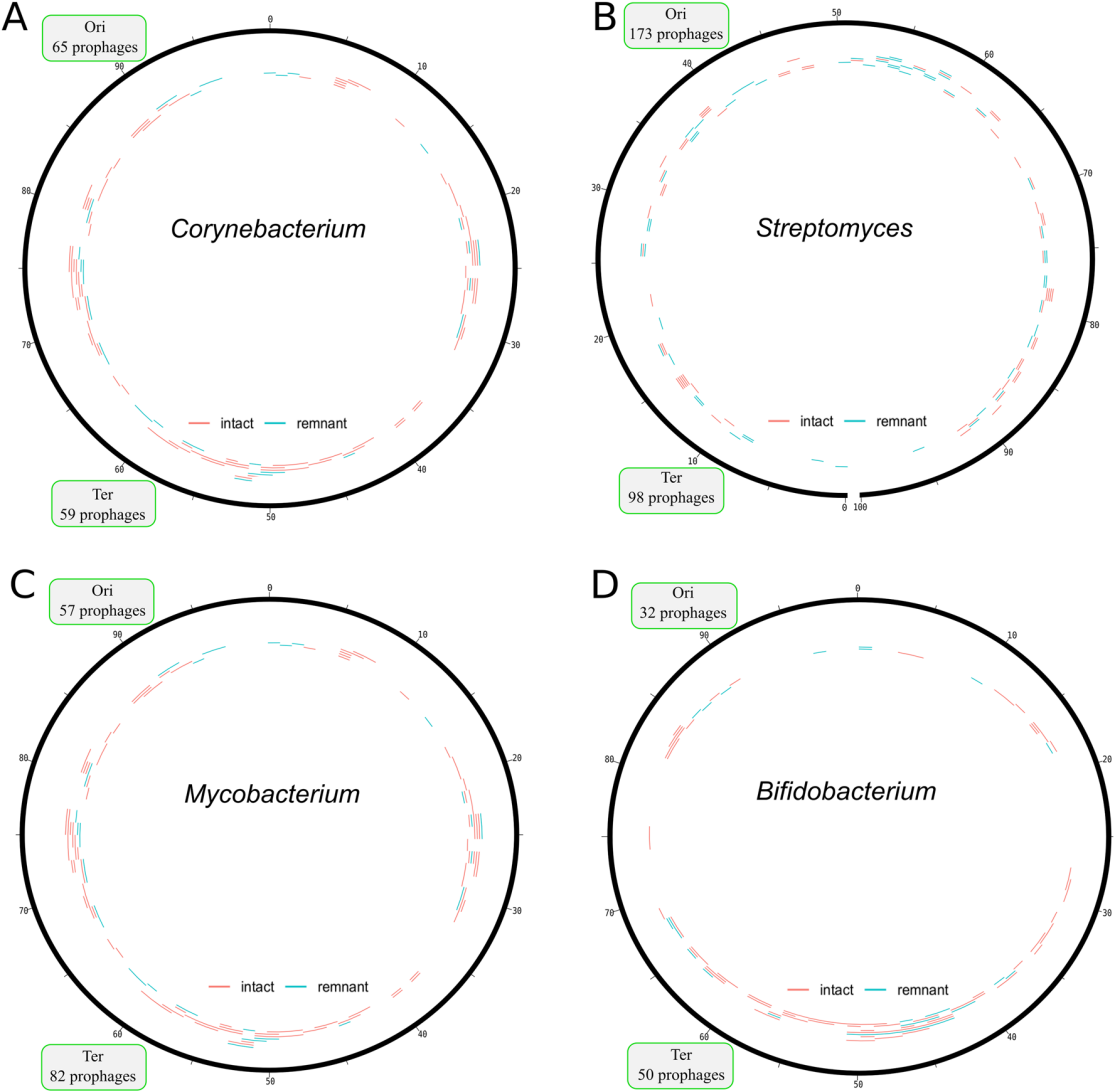
Prophage distribution across the *Corynebacterium*, *Mycobacterium, Bifidobacterium* and *Streptomyces* genomes. Prophage location, classification (Red = Intact and blue = remnant) and their approximate size is displayed inside the circular plot. The numbers account for identified prophages in the upper (Ori, 0–25 and 76–100, 26–75 for *Streptomyces*) and lower half (Ter, 26–75 and 0–25, 76–100 for *Streptomyces*) of the chromosome.

As a next step, we investigated the genomic localization of prophage elements relative to the origin of replication. Here, we focused on four important actinobacterial genera, namely *Corynebacterium*, *Mycobacterium*, *Bifidobacterium* and *Streptomyces*, representing the largest fraction of sequenced genomes (Supplementary Table S1, Sheet3). The genomic positions of all prophages were normalized according to their relative orientation to the origin of replication (of note, *Streptomyces* have linear chromosomes with the *oriC* at the center). The redundancy of highly similar genomes was removed by choosing one representative prophage sequence for each assigned cluster based on the abovementioned clustering approach (Supplementary Table S4). The resulting analysis showed that several different prophages are clustered in hotspots or are tandemly duplicated at a specific location (Fig. 4). For example, *Corynebacterium* showed a hotspot of intact prophages at 5–10 bp % and a higher density of intact and remnant prophages at 60–80 bp %. However, this may also be due to sequencing bias caused by overrepresentation of single species in databases.

As a general trend, we observe a larger fraction of prophages at the region of the terminus. This overall trend is in agreement with previous studies^8,9,42^, but the reason for this trend has—to the best of our knowledge—not been investigated so far. One possible explanation could be based on gene dosage effects of replicating genomes. During multi-fork replication, genes close to the origin of replication have a higher gene dosage than genes close to the terminus. Previous studies found indeed a close correlation between gene dosage and transcript levels, with genes at the *ori* showing on average higher transcript levels^43^. In the interest of a prophage, hiding in a bacterial chromosome, a position close to the “more silent” terminus might promote host tolerance and subsequent domestication. Previous studies have underlined the importance of efficient silencing of prophage elements to repress spontaneous prophage induction. In *C. glutamicum* the Lsr2-like xenogeneic silencer protein CgpS was shown to be crucial for the silencing of the large prophage region CGP3, which is also located close to the terminus^27^. Already minor reduction of CgpS levels resulted in a strong increase of CGP3 induction. The expression level of *cgpS* is very high, even in the dormant state of the prophage, and is subject to positive autoregulation^44^. It is likely that localization of CGP3 close to *ori* would require even higher levels of the silencer protein. Therefore, the need for efficient phage gene silencing might be one reason for the accumulation of prophage elements near the terminus, but requires further experimental investigation.

Exact prophage integration sites where further mapped by analyzing 2 kb upstream and downstream genomic regions on both sides of identified prophages genomes (Supplementary Table S6). Overall, we found the integration of prophages near tRNA genes at least for 15% of total analyzed loci, which is in line with the previous findings (Supplementary Table S6)^45,46^. The presence of the tRNA gene has been suggested to be utilized by phage elements in host genomes as the alternate integration attachment sites in case if preferred loci is mutated or when it is already occupied by other phages^47^.

### Genomic content of prophages

**Figure 5:**
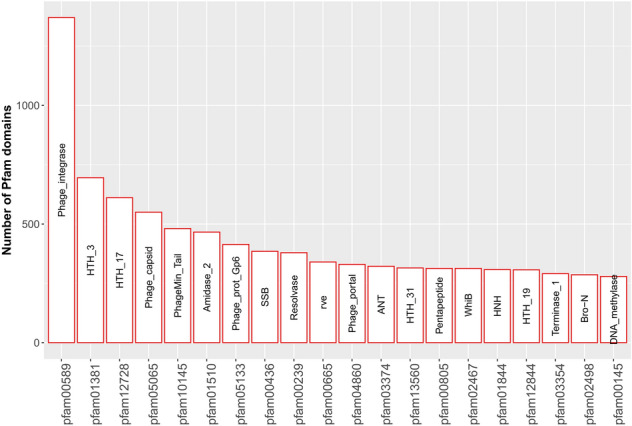
Distribution of unique COG protein domains in prophages. The top 20 most abundant protein (pfam) domains encoded by the identified prophage genomes.

In the following, we further analyzed conserved domains using the Pfam database (Fig. 5, Supplementary Table S7). The pfam family ‘phage integrase’ (pfam00589) represents the most frequently discovered genes in our dataset (Fig. 5). Additionally, ‘rve’ (pfam00665) domains are known to belong to the same group of integrase proteins. We furthermore find a high abundancy of genes containing the Pfam domains HTH_3 (pfam01381) and HTH_17 (pfam12728) belonging to phage repressors like CI/Cro, originally described from *E. coli* (pro)phage Lambda^48^ or XRE, which was discovered as regulator of *B. subtilis* (pro)phage PBSX^49^. Among the top 20 hits, we also found the ‘ANT’ (pfam03374) domain, which also referred to as ‘KilAC’ domain found in phage antirepressors^50^. Another important part is made up by phage structural proteins belonging to the categories ‘phage capsid’ (pfam05065), ‘phage minor tail protein’ (pfam10145), and ‘phage portal protein’ (pfam05133 and pfam04860) (Fig. 5). A previous study of our group identified WhiB- (pfam02467) and Lsr2-type (pfam11774) regulators as most abundant transcriptional regulators encoded by actinobacteriophages genomes^51^. This is in line with the analysis of prophage genomes, where we found WhiB among the top twenty domains (Lsr2 at position 80, see Supplementary Tables S7, S8). While WhiB represents the most abundant transcriptional regulator encoded by phage genomes, in prophage genomes the HTH_3 and HTH_17 domains found in cro/CI proteins show a higher abundancy. Previous studies also provided striking evidence for mutual interaction between the prophage and the host showing that prophages provide beneficial functions promoting stress tolerance, virulence or antiviral defense. However, those are likely specific to the particular host and are not found among the most abundant categories^52,53^.

In addition to single genes, the Gene Ontology (GO) database^54^ spans defined categories related to their corresponding biological function. We explored this GO annotation associated functional categories mapped on each unique Pfam protein domains to understand the difference between the known actinobacteriophages and the identified prophage genomes. The comparative analysis based on the top 20 most abundant GO annotations revealed significant differences between the phage and prophage genomes (Supplementary Fig. S4). As expected, prophage sequences showed high enrichment in DNA integrase-related protein domains. In addition, the group of proteins showing ‘DNA-binding transcription factor activity’ represents one of the top 20 functional categories in prophages.

### Prophage-encoded antiphage defense systems and biosynthetic gene clusters

Actinobacteria genomes contain various biosynthetic gene clusters (BGCs) involved in the synthesis of specialized metabolites. Recent studies have shown that secondary metabolites belonging to the group of anthracyclines^55^ or aminoglycosides^56^, produced by *Streptomyces*, play a role in the chemical defense against phage infection. Molecules of these compound classes were shown to inhibit phage infection in widely divergent bacterial hosts by blocking an early stage of the phage infection cycle. Consequently, we systematically searched for BGCs in prophage elements using antiSMASH^57^. This analysis revealed the presence of small, putative BGCs in only 0.6% (13/2112) of the total analyzed genomes (Supplementary Table S9). In particular, several clusters predicted to be involved in the synthesis of class I lanthipeptides were found in prophages of *Streptomyces,* making them interesting candidates for further experimental validation. The identified BGCs are discovered among both intact and remnant phages. Type III polyketide synthases (T3PKS) were exclusively found in prophages detected from plasmid genomes.

**Figure 6:**
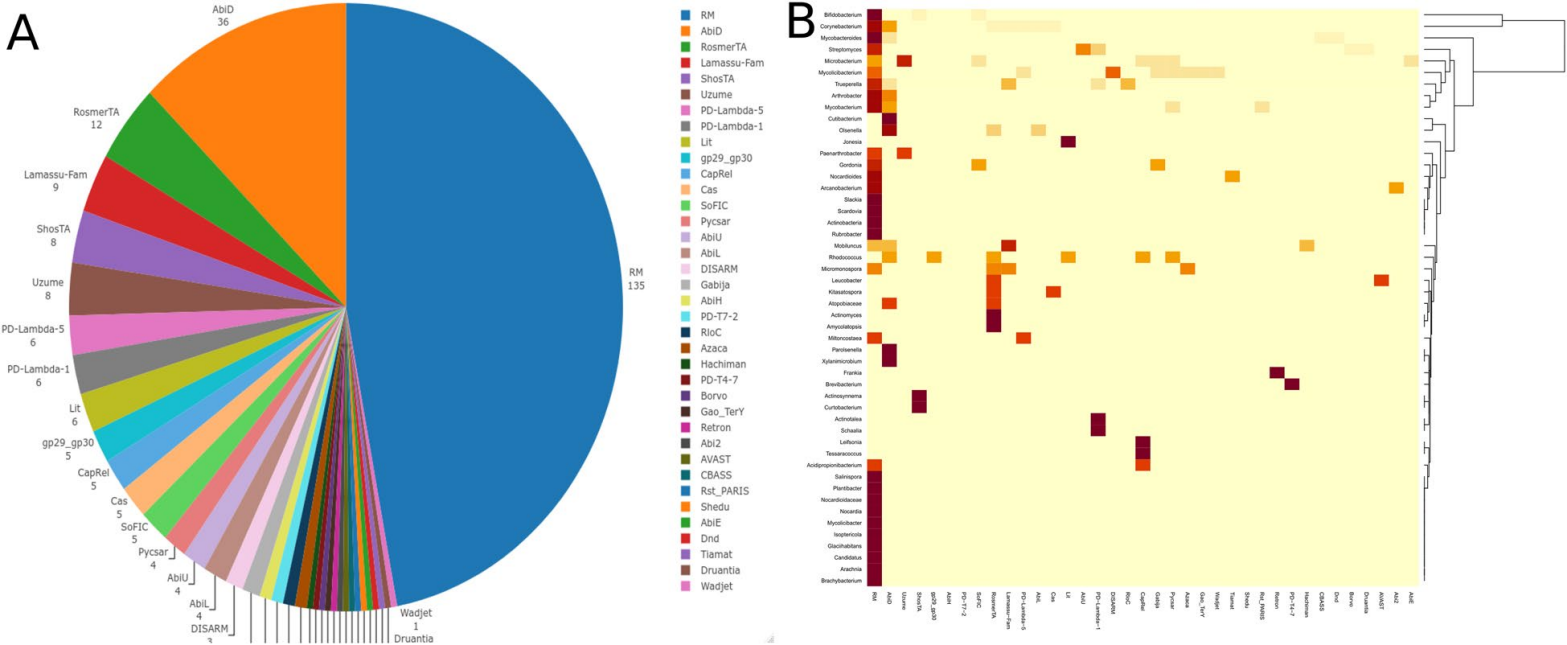
Distribution of defense systems. (**A**) The pie chart represent the distribution and abundance of 37 defense systems identified within prophage genomes. (**B**) The heatmap represents the distribution of prophage encoding 37 defense systems according to the host genus.

Previous studies also revealed that prophages may be important carriers of genes involved in phage defense. Here, we looked for potential antiviral systems within identified prophage genomes using the DefenseFinder tool^58^. The resulting analysis discovered the presence of 37 different defense systems within 10% (227/2112) of total analyzed prophage genomes (Fig. 6A, Supplementary Table S10). Unsurprisingly, RM (restriction-modification) systems make up the largest fraction, followed by AbiD (abortive infection)^59^, RosmerTA, Lamassu-Fam, ShosTA, Uzume, Lit (mRNA decay and inhibition of translation)^60^, and CRISPR/Cas systems^61^. As expected, most identified defense systems display a specific distribution pattern according to the host genus (Fig. 6B). The analyses focusing on plasmid prophages revealed at least eight different types of defense systems (AbiD, AbiE, Cas, dCTPdeaminase, Lamassu-Fam, RosmerTA, ShosTA and RM) present within 23% (36/152) of the analyzed genomes (Supplementary Table S10). Interestingly, in host genera where prophages not encode RM systems, like *Rhodococcus*, *Frankia* or *Leucobacter*, other defense systems are found on prophages instead (AbiD, Retron and AVAST, respectively).

Overall, the data suggest a significant contribution of prophage genomes in phage defense. However, we did not find any evidence for large BGCs involved in the synthesis of known antiphage molecules to be located on prophage islands. This is in line with previous studies showing that large BGCs are rarely found in temperate phages or prophages^62^.

## Conclusions

In this study, we performed a systematic analysis of prophage elements in actinobacterial genomes using the prophage prediction tool VIBRANT. Overall, this approach resulted in the prediction of more than 2000 putative prophage elements found in approx. 50% of actinobacterial genomes. This appears as a rather low fraction of lysogenized strains in comparison to other genera (e.g. 99.5% for *Acinetobacter baumanii*^35^ and > 90% in *Streptococcus pyogenes*^8^) and it cannot be ruled out that several elements might have been missed in our analysis. However, the prediction tool VIBRANT was among the top performing prediction tools in terms of accuracy and precision in recent comparative analysis conducted on a set of manually curated genomes^29^. On average, prophage elements were predicted to account for 1% of the genomes, with some cases going up to 10%, which is in line with previous studies^9^. Our analyses suggested furthermore a high fraction of remnant elements, which are probably defective in some or several functions. However, previous studies demonstrated important physiological or regulatory functions of cryptic prophages being involved in stress tolerance^6^, microbial warfare^10,62^ or by functioning as regulatory switches^63^. Also for cryptic prophages, induction may still be possible and spontaneous induction events of prophages were shown by several studies to significantly shape bacterial physiology and host-microbe interactions, e.g. by promoting the formation of biofilms or the release of toxins^15^. Therefore, our study provides an important basis for further functional analysis.

Recent years have seen a dramatic increase in the availability of actinobacteriophages genomes. This is mainly thanks to the enormous effort of educational programmes, like SEA-PHAGES^21^. Given the high diversity of sampled actinobacteriophages, which is now available at the Actinobacteriophage database (https://phagesdb.org/), the diversity of prophage elements in actinobacterial genomes is even more remarkable. In our analysis, only 1.3% of the predicted prophage elements cluster with known actinobacteriophages. These results highlight the enormous phylogenetic diversity of prophage elements “sampled” by their bacterial hosts in the course of evolution. Altogether, these results underline the importance of including this information for studying phage biology in this large bacterial phylum.

## Material and methods

### Genomic data set

Complete genome sequences (2406 chromosomes and 844 plasmids) belonging to the phylum Actinobacteria were downloaded from the NCBI FTP site (https://www.ncbi.nlm.nih.gov/genome/browse#!/prokaryotes/) on 26 August 2021 (Supplementary Table S1, Sheet1–2). In addition, 3433 actinobacteriophages sequences with valid NCBI accession numbers list from PhagesDB^18^ were downloaded using NCBI-genome-download python script (https://github.com/kblin/ncbi-genome-download) on 13 August 2021.

### Detection of prophage-like sequences in the actinobacterial genomes

The putative prophage-like sequences were predicted in the actinobacterial strains and associated plasmid genomes based on machine learning approach using Virsoter2 v.2.2.3^25^ and VIBRANT (Virus Identification By iteRative AnnoTation) v1.2.1^26^ with the default parameters. Predicted prophages from both programs were separately analyzed to remove unwanted host contamination and ensure completeness of viral sequences using CheckV v0.8.1^64^.

### Annotation and classification

All 2112 prophage nucleotide sequences were annotated based on conserved protein domains using RPS-TBLASN^65^ against the Conserved Domain Database (CDD) database^34^. In the resulting investigation, six short sequences that do not encode any conserved protein domains were excluded from the further analysis. Additionally, gene-level prediction and annotation were performed using Prokka version 1.11^66^ against the customized databases (pVOGs—Prokaryotic Virus Orthologous Groups^67^, CDD—Conserved Domain Database^34^, and NCBI viral proteins). The prophage was considered "intact" if the sequence encoded an integrase domain and one or more structural PFAM conserved protein domains (Capsid, Tail, and portal) collected from manual curation and using PHROG database^68^ (Supplementary Table S11). However, if the prophage was lacking an integrase domain, the element was considered a "remnant or defective". Pfam2go file version date: 2022/01/15 were downloaded for mapping of Pfam associated GO terms^54^ from the Gene ontology website (http://current.geneontology.org/ontology/external2go/pfam2go).

### Integration analysis

The most abundant prophage encoding three host genus (*Mycobacterium, Corynebacterium, Bifidobacterium* and *Streptomyces*) strains were used for the density distribution analysis. Initially, identified prophages were normalized based on their identified coordinate positions divided by the total size of host strain chromosomal sequences. Subsequently, the distribution of prophages on the host reference chromosome was displayed using the R package “ggbio version 1.34.0”^69^ and the Artemis with DNAPlotter program^70^. The 2 kb upstream and downstream genomic fasta of the prophage element were extracted from the bacterial genome using the list of detected prophage coordinates using a custom bash script. Further, resulting flanking sequences from each side were annotated using Prokka v.1.11^66^ and CDD database^34^.

### Detection of defense genes and secondary metabolite clusters

Complete proteome sequences of identified prophage elements were screened for anti-phage systems using the DefenseFinder tool with default setting^58^. Potential biosynthetic gene clusters were identified using the antiSMASH 6.0.1 tool with default parameters^57^.

### Clustering and phylogenetic tree based on genome-based similarity

The prophage and actinobacteriophage nucleotide sequences were clustered based on genome-wide average nucleotide identity (ANI) with default parameters (95% ANI and 80% coverage) using a Perl script ClusterGenomes (https://github.com/simroux/ClusterGenomes). Additionally, phylogenetic-like trees were constructed based on the genome-wide nucleotide k-mer frequency distribution. The output frequency matrix was used to calculate the pairwise distance between the genomes using the Jensen–Shannon divergence method^71^. Further, clustering was performed to generate the phylogenetic tree was using the following approach based on R (https://bioinformaticshome.com/bioinformatics_tutorials/R/phylogeny_estimation.html). Finally, the phylogenetic tree was displayed using ggtree ‘3.3.1′^72^ in R. Prophage and phage genomic similarity were compared and visualized using clinker and clustermap.js^73^.

### Data visualization and statistical analysis

All data-set visualization and statistical analysis were conducted using the following R packages (gplots v. 3.0.1.2, ggplot2 v. 3.2.1^74^, rstatix v. 0.6.0, ggpubr v. 0.4.0, and, tidyverse v. 1.3.0).

## Supplementary Information

Supplementary Information 1.

Supplementary Information 2.

## Data Availability

The bacterial genomes used in the study can be downloaded from NCBI FTP SITE using the unique accession numbers provided in Supplementary Table S1. The predicted prophages using the insilico programs and custom scripts used for the analysis are available on Github (https://github.com/sharmavikas3529/Prophage-genomics.git).
