# Supplementary material for: Systematic analysis of prophage elements in actinobacterial genomes reveals a remarkable phylogenetic diversity
Source: Sci Rep. 2023 Mar 17;13:4410. doi: 10.1038/s41598-023-30829-z (PMC10023795; doi:10.1038/s41598-023-30829-z)
Supplement: Supplementary file 1 — Supplementary Information 1. [file 41598_2023_30829_MOESM1_ESM.pdf]

Supplementary information to:

Systematic analysis of prophage elements in actinobacterial genomes reveals a remarkable phylogenetic diversity

Vikas Sharma[1], Max Hünnefeld[1], Tom Luthe[1], and Julia Frunzke[1]

[1]Institute of Bio- und Geosciences, IBG-1: Biotechnology, Forschungszentrum Jülich, 52425 Jülich, Germany

*Corresponding authors:

Vikas Sharma; Email: v.sharma@fz-juelich.de; Phone: +49 2461 612544

Julia Frunzke; Email: j.frunzke@fz-juelich.de; Phone: +49 2461 615430

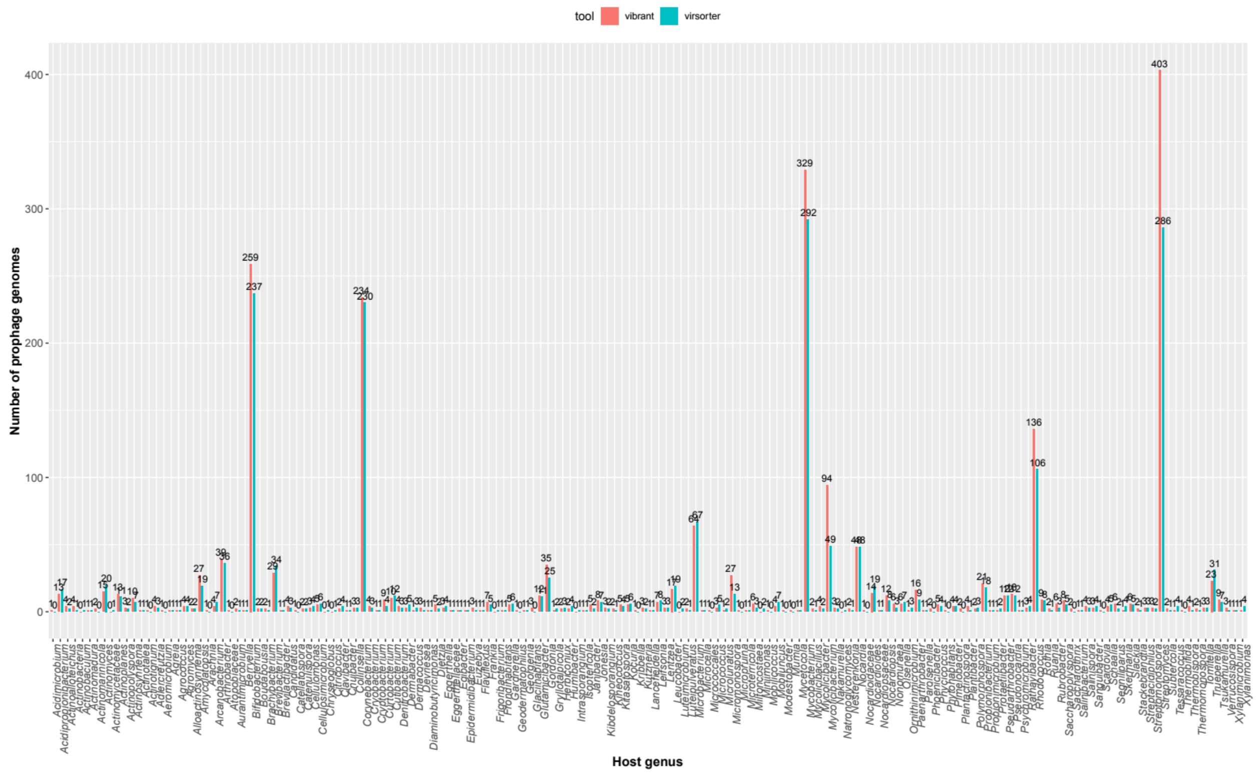

Figure S1: Comparison between prophage prediction programs. Bar plot represents differences in the number of prophages predicted by Virsorter2[1] and Vibrant[2] according to host genus.

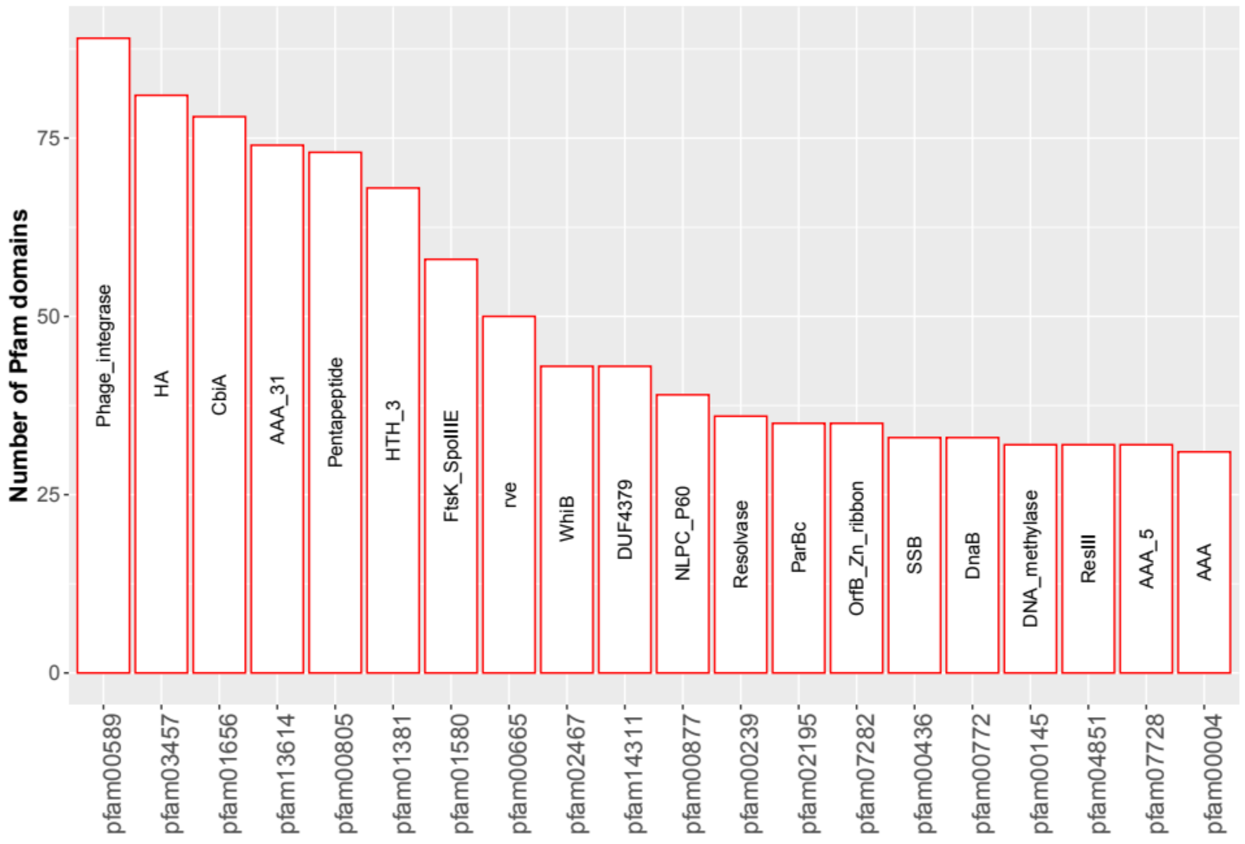

Figure S2: Distribution of Pfam domains in plasmid prophages. The 20 most abundant Pfam domains were detected in the prophages from plasmid genomes.

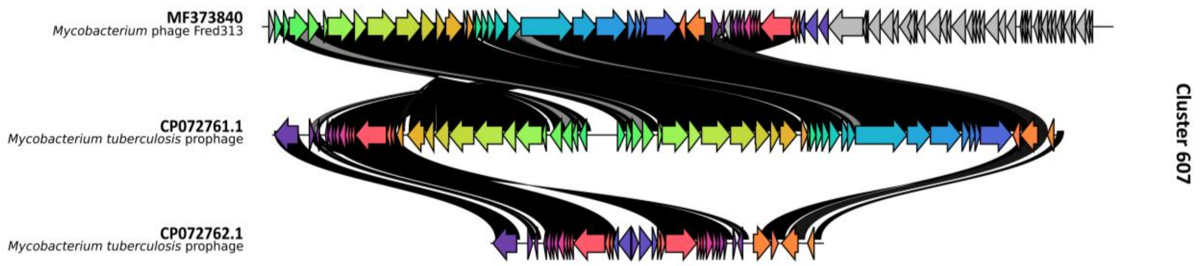

Figure S3: Syntenic analysis between prophages and *Mycobacterium phage Fred313***.** Shared genes between the prophages and known Mycobacterium phage. Arrows of the same color indicate genes that are shared in the genomes. The grey horizontal bar represents the level of similarity between the genes across the compared genomes.

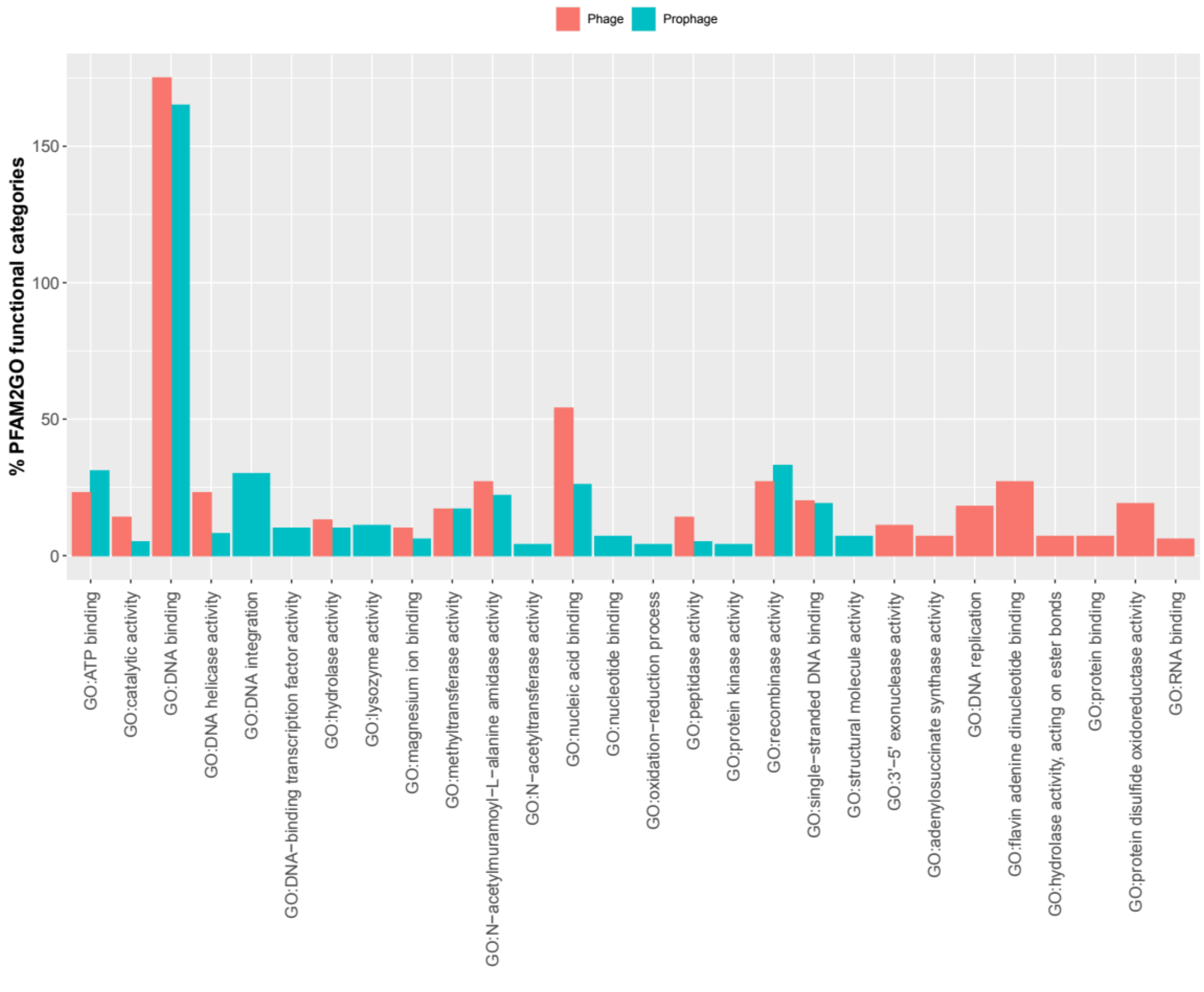

Figure S4: Pfam2go functional categories comparison. The barplot represents the top 20 enriched functional categories across the prophage and actinobacteriophage genomes.

REFERENCES

1. Guo, J. *et al.* VirSorter2: a multi-classifier, expert-guided approach to detect diverse DNA and RNA viruses. *Microbiome* **9**, 1–13 (2021).

2. Kieft, K., Zhou, Z. & Anantharaman, K. VIBRANT: Automated recovery, annotation and curation of microbial viruses, and evaluation of viral community function from genomic sequences. *Microbiome* **8**, 1–23 (2020).
